# Metronomic Capecitabine in Patients With Hepatocellular Carcinoma Unresponsive to or Ineligible for Sorafenib Treatment: Report of Two Cases

**DOI:** 10.5812/hepatmon.11721

**Published:** 2013-09-09

**Authors:** Sara Marinelli, Alessandro Granito, Fabio Piscaglia, Matteo Renzulli, Angela Stagni, Luigi Bolondi

**Affiliations:** 1 Department of Medical and Surgical Sciences, S.Orsola-Malpighi Hospital, University of Bologna, Bologna, Italy; 2 Radiology Unit, University of Bologna, S.Orsola-Malpighi Hospital, Bologna, Italy

**Keywords:** Hepatocellular Carcinoma, Capecitabine, Sorafenib

## Abstract

**Introduction:**

Sorafenib, an oral multikinase inhibitor, is the only systemic agent proven to be effective in patients with hepatocellular carcinoma (HCC). There are no approved second line systemic therapies in patients who have had disease progression on or are not eligible to sorafenib.

**Case Presentation:**

We describe two cases of unresectable HCC that were treated with low, "metronomic" doses of capecitabine. In the first patient, capecitabine was used after sorafenib failure. In the second case, treatment with capecitabine was attempted since the patient was considered not eligible for sorafenib due to spontaneous hepatic bleeding of a large HCC lesion. Treatment was effective and well tolerated in both patients with long-lasting objective responses.

**Conclusions:**

Lacking established second-line therapy, metronomic capecitabine may be a valid alternative in the treatment of HCC patients who are judged not eligible for sorafenib or those having progression disease on sorafenib.

## 1. Introduction

Hepatocellular carcinoma (HCC) is the sixth most common neoplasm and the third most frequent cause of cancer-related death worldwide (1). Curative treatments for early-stage tumors include liver transplantation, resection and percutaneous ablation. However, the majority of patients are not eligible for curative therapies because of tumor extent or underlying liver dysfunction (2). Sorafenib (Nexavar; Bayer HealthCare Pharmaceuticals, Montville, NJ, USA), an oral multi-kinase inhibitor, is presently the only effective therapy in the advanced stage of HCC. In two randomized phase-III studies, Sorafenib has been shown to increase the mean survival time by approximately 3 months (3, 4). These data established sorafenib as the reference standard systemic treatment for patients with advanced HCC who still have preserved liver function (2).

Following the approval of sorafenib, several phase III studies with new drugs are ongoing to assess other molecularly targeted agents that inhibit different pathways of hepatocarcinogenesis (4). Pending the results of ongoing studies, no second-line treatments currently exist outside of clinical trials for HCC patients who are resistant or intolerant to sorafenib. We describe two cases of unresectable advanced HCC referred at the Department of Medical and Surgical Sciences of the University of Bologna who obtained long-lasting objective responses after treatment with metronomic capecitabine. Their unexpectedly good responses and the personalized management of therapy can provide suggestions to optimize the treatment of HCC when sorafenib fails or is not indicated.

## 2. Case Presentation

### 2.1. Case 1 Description

This case refers to a 53-year old woman with HCV-related cirrhosis and portal hypertension, not included in any surveillance program. In October 2011, due to abdominal pain she underwent abdominal ultrasound (US) and contrast-enhanced computed tomography (CT), both showing six focal liver lesions suspicious for HCC, the largest in the right lobe and measuring 70 mm × 87 mm. In November 2011 she was admitted to her local hospital because of hepatic decompensation with ascites and endoscopic finding of grade III esophageal varices at high risk of bleeding. She was referred to our Unit in order to confirm the suspicion of HCC. Contrast-enhanced ultrasound (CEUS) of focal liver lesions was consistent with the diagnosis of multifocal HCC. A biopsy specimen obtained from the major nodule showed HCC (Edmondson grade II). Due to the detection on Doppler US of a high flow arteroportal fistula within the major HCC lesion, she underwent percutaneous transcatheter endovascular embolization. Control endoscopy showed grade I-II esophageal varices. Since the patient was considered not eligible for endovascular treatment of HCC, but had a good liver function (Child-Pugh class A), treatment with sorafenib was started in December 2011. Alpha-fetoprotein (AFP) serum levels were 19 ng/ml (< 7 ng/ml).

**Figure 1.**
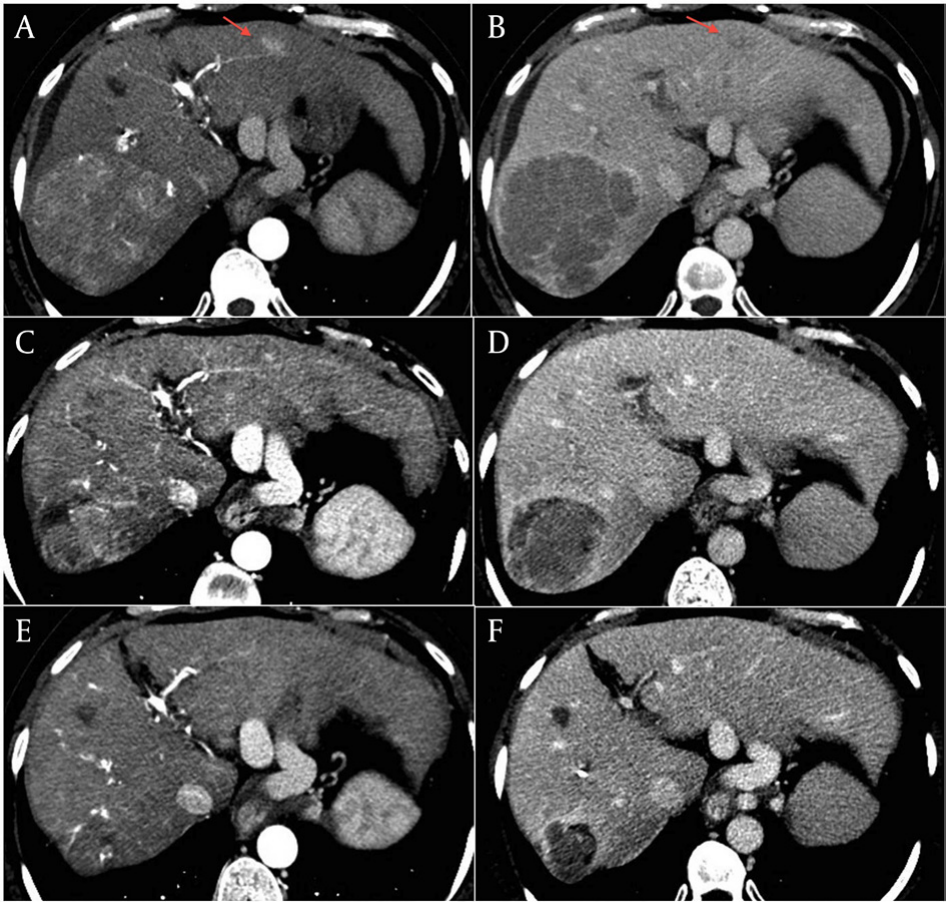
Contrast-enhanced CT scan of the liver performed as baseline assessment in March 2012 (A and B), at the end of June 2012 (C and D), and in January 2013, showing a progressive shrinkage of the the large HCC lesion of the right lobe. A, C, and E panels: arterial phase. B, D, and F panels: equilibrium phase Note that the HCC lesion detected in the segment II (arrows in panels A and B) was also markedly reduced at the first CT control (C and D), and was no longer clearly distinguishable from the surrounding tissue at the last radiologic control (E and F).

Three months later, treatment was stopped due to CT evidence of disease progression. The major lesion had increased to 90 mm × 87 mm, with persistent high vascularization, and involved the corresponding segmentary portal vessel (Figure 1, A and B). There was also an increase in size and in number of the other lesions from 5 to 7. AFP serum levels were 17 ng/ml. Given the absence of second-line therapy and the unmodified liver function, we proposed treatment with capecitabine. The patient gave her informed consent and in March 2012 she started therapy at a dosage of 500 mg twice/daily. Since one month later she developed a grade 3 hand-foot syndrome (HFS), treatment was temporarily stopped and she was treated with emollient and urea-based creams until complete resolution of the HFS. Capecitabine was re-started at a reduced dose of 750 mg per day but, in June 2012, grade 2 HFS and anemia (hemoglobin dropped from 12 to 9 g/dl) occurred. Capecitabine was again interrupted, until resolution of HFS and anemia, and then re-started at a dosage of 150 mg thrice daily. At the end of June, contrast-enhanced CT scan showed size reduction of the major lesion to 75 mm × 72 mm with hypodense necrotic areas (Figure 1, C and D), the persistence of three subcentimetric lesions, and the complete disappearance of the other four lesions. Capecitabine was continued and, after a further dose reduction to 150 mg twice daily due to a transient reappareance of grade 2 HFS which required one week interruption, was well tollerated. The last contrast-enhanced CT assessments showed further decrease of the tumor, with the major lesion decreased to 43 mm × 39 mm in October 2012, and to 39 mm × 39 mm in January 2013 (Figure 1, E and F). AFP serum levels remained stable over the treatment period. The patient is still in good clinical condition (ECOG performance status 0) with a preserved liver function.

### 2.2. Case 2 Description

**Figure 2.**
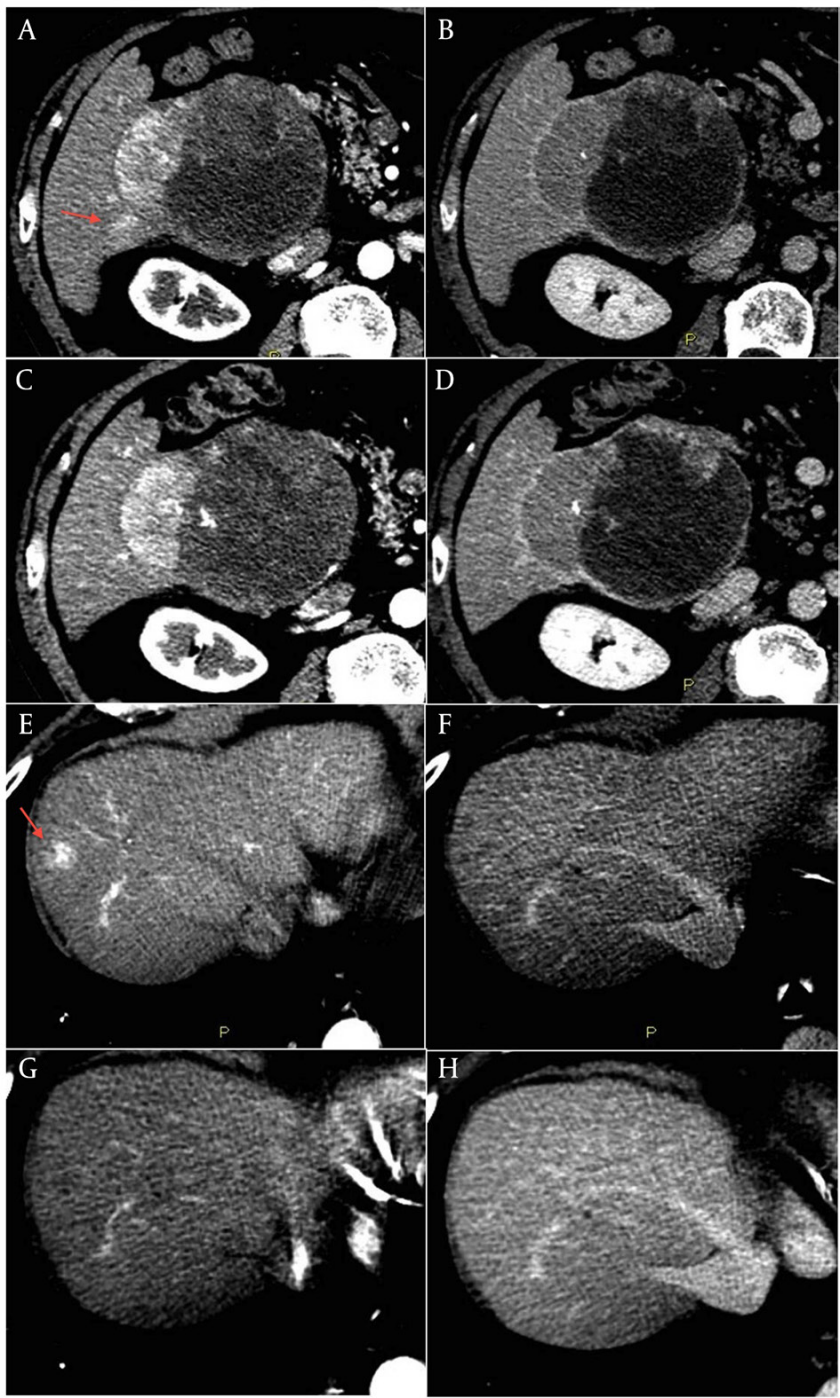
Contrast-enhanced CT scan of the liver performed in December 2011, as baseline assessment, showing the large HCC lesion of the right lobe including a necrotic component (A and B), a small satellite lesion (arrow in panel A), and the lesion in segment VIII (arrow in panel E). A, C, E, and G panels: arterial phase. B, D, F, and H panels: equilibrium phase. The last contrast-enhanced CT scan performed in January 2013 showed a reduction in vascularity of the large lesion with an increase of tumor necrosis, and a significant shrinkage of the satellite lesion (C and D). The lesion in segment VIII was no longer clearly distinguishable from the surrounding tissue (G and H).

This is the case of an 80-year old male without history of liver disease. His past medical history included hypertensive heart disease and chronic atrial fibrillation with cardioembolic cerebellar stroke in June 2009 for which he was assuming warfarin. In May 2010 the patient was admitted for severe anemia due to acute bleeding of a voluminous mass of the right liver lobe. Anticoagulant therapy was stopped and he underwent a contrast-enhanced liver CT that showed a radiologic pattern consistent with a malignant lesion. A percutaneous biopsy of the liver lesion revealed grade 2 HCC. AFP serum levels were normal. In December 2011 the patient was referred to our Unit and was staged with a contrast-enhanced CT scan that showed a lesion in segment VI of 111 mm x 95 mm with a small adjacent satellite lesion, and other three lesions with a maximum diameter of 22 mm (segment I), 25 mm (segment VIII), and 24 mm (segment IV) (Figure 2, A, B, E, F). Due to the multifocal HCC with a high risk of bleeding and spontaneous rupture of the major lesion, we judged the patient not eligible for endovascular or sorafenib treatment (5). Considering the absence of chronic liver disease, and the good clinical conditions (ECOG Performance status 0), we proposed an off label treatment with metronomic capecitabine at the dose of 500 mg thrice daily. The patient gave the informed consent and started treatment in January 2012. The contrast-enhanced CT scan, scheduled every three months, showed a progressive reduction in vascularity of all nodules with a global dimensional stability of the major lesion. The last control CT scan of January 2013 showed an increase of necrotic area within the largest lesion (Figure 2, C and D). The lesion of the segment VIII (Figure 2, G and H), after a reduction in size over the time, was no longer distinguishable from the surrounding tissue. The patient is still in treatment with a good quality of life, without side effects or laboratoristic alterations.

## 3. Conclusions

Sorafenib represents a breakthrough in the treatment of HCC, and proves that molecular targeted therapies can be effective in this complex disease. However, aside from best supportive care, at present there is no established second line therapy for patients refractory or intolerant to sorafenib. Conventional chemotherapy is not used since HCC arises in the majority of cases in a context of chronic liver disease which can influence drug metabolism and enhance their toxicity. Capecitabine is an oral 5-fluorouracil (5-FU) prodrug that is absorbed in the intestine. It is then metabolized to FU in three-step enzymatic reaction, the final one being the conversion in the liver and in the tumor by thymidine phosphorylase (TP). TP is present at higher levels in tumor cells compared to healthy tissue, allowing a selective activation of the drug (6). Capecitabine is currently used in the treatment of metastatic colorectal and breast cancer at the dosage of 1250 mg/m^2^ twice per day for two weeks followed by a one-week rest period. The most common adverse events (AEs) are hyperbilirubinemia, diarrhoea and HFS. Other frequent AEs are also fatigue, anaemia, abdominal pain and nausea (6). Cardiac toxicity is a potential AE whose mechanism is unknown but it is proposed to be secondary to myocardial ischemia induced by coronary vasospasm (7).

In the last years, the concept of “metronomic” chemotherapy has been introduced in oncology. It is based on the chronic administration of chemotherapeutic agents at relatively low, minimally toxic doses, and with no prolonged drug-free breaks in order to optimize antiangiogenic properties of the drugs and to reduce toxicities (8, 9). This treatment regimen may be particularly appropriate in patients with mild impairment of liver function, as in the case of compensated cirrhosis. Capecitabine is reported to be used both in advanced HCC and as postoperative adjuvant therapy after curative resection. Treatment resulted to be safe in patients with cirrhosis, in particular at metronomic dosage (10-12). While standard schedule of capecitabine in cirrhotic patients can deteriorate liver function, increasing bilirubin or inducing ascites, metronomic dosage seems to increase tolerability and to reduce the risk of liver function deterioration. Furthermore the metronomic schedule does not seem to affect the activity of the drug (10, 12, 13). The definition of the optimal metronomic dosage of chemotherapics is a very important issue. It was proposed that the metronomic dose should be the highest one within a metronomic schedule, which does not induce clinical bone marrow perturbation since bone marrow suppression acts as a proangiogenic stimulus (14). In our patients we used tailored treatment schedules with a higher daily dosage in the patient without liver disease.

In conclusion, in the patients here presented, treatment with metronomic capecitabine obtained unexpectedly good therapeutic efficacy. Although there are no controlled clinical trials on the efficacy of metronomic capecitabine in patients with HCC, in the absence of established second-line therapies, this treatment might be considered in patients unresponsive or intolerant to sorafenib, or in those who are not eligible for sorafenib because of contraindications.
